# In Situ Harvesting and Molecular Identification for the Germinating Species Diversity of Dinoflagellate Resting Cysts in Jiaozhou Bay, China

**DOI:** 10.3390/life15111670

**Published:** 2025-10-27

**Authors:** Shuo Shi, Wanli Yang, Zhe Tao, Fengting Li, Ben Wei, Caixia Yue, Yunyan Deng, Lixia Shang, Zhaoyang Chai, Ying-Zhong Tang

**Affiliations:** 1 CAS Key Laboratory of Marine Ecology and Environmental Sciences, Institute of Oceanology, Chinese Academy of Sciences, Qingdao 266071, China; 2 University of Chinese Academy of Sciences, Beijing 100049, China; 3 Shandong Key Laboratory of Marine Ecological Restoration, Shandong Marine Resource and Environment Research Institute, Yantai 264006, China; 4 China Laboratory for Marine Ecology and Environmental Science, Qingdao Marine Science and Technology Center, Qingdao 266237, China; 5 Qingdao Innovation and Development Center, Harbin Engineering University, Qingdao 266000, China; 6 Animal, Plant and Food Inspection Center of Nanjing Customs, Nanjing 210000, China

**Keywords:** in situ monitoring of germination, dinoflagellate, resting cyst, Germlings Harvester (GEHA), HABs

## Abstract

Dinoflagellate resting cysts are critical to dinoflagellate ecology, acting as a key seed source for initiating harmful algal blooms (HABs) through their germination. However, the in situ germination dynamics of these cysts remain poorly understood due to technical challenges. To overcome this, we utilized the Germlings Harvester (GEHA), an in situ germination device we designed, to collect water samples containing dinoflagellate cysts germinated from marine sediments in Jiaozhou Bay, China, after 5 and 20 days of incubation. By combining the GEHA with metabarcoding analysis targeting 28S rDNA-specific primers for dinoflagellates, we identified 44 dinoflagellate species spanning 31 genera, 18 families, and 7 orders. Of these, 12 species were linked to HABs or recognized as toxic, including *Azadinium poporum*, *Alexandrium leei*, *Alexandrium pacificum*, *Akashiwo sanguinea*, *Karlodinium veneficum*, *Stoeckeria algicida*, and *Luciella masanensis*. Additionally, five species were newly identified as cyst producers, and one symbiotic dinoflagellate, *Effrenium voratum*, was detected. Our results also found that germinated dinoflagellate species increased from 23 to 34 with extended incubation, and the ratio of mixotrophic to heterotrophic species was approximately 2:1 in the samples of in situ sediments and seawater outside GEHA, as well as across germination durations (Sg-5 d vs. Sg-20 d). These findings provide essential field evidence for the role of resting cysts in driving HAB formation in this region and highlight the efficacy of the GEHA-based approach for studying in situ cyst germination dynamics, offering a robust tool for monitoring, early warning, prevention, and forecasting of HABs.

## 1. Introduction

Dinoflagellates, pivotal primary producers in marine ecosystems, are also major contributors to harmful algal blooms (HABs) globally. Among HAB events, about 75% of them are caused by dinoflagellates [1], and dinoflagellates account for 40% of the species forming HABs globally [2]. In China, dinoflagellate blooms frequently occur in coastal waters, spanning the Bohai, Yellow, East China, and South China Seas. These blooms cause tremendous damage to fisheries, mariculture, tourism, and human health, as well as marine ecosystems [3]. The official report “Bulletin of China Marine Disaster” from 1989 [4] and several recent reviews [3] state that among the 17 major large-scale HAB-causing species in China’s coastal waters, 9 are dinoflagellates, accounting for approximately 53%. Of these, five species (e.g., *Gymnodinium catenatum* and *Karenia mikimotoi*) have caused mortality in marine animals or even humans due to bioaccumulation of toxins and transmission through the food chain [3].

About 200 out of approximately 2900 extant dinoflagellate species have been reported to produce resting cysts, and more than 20 of them form HABs [5,6]. The formation, dormancy, and germination of resting cysts are ecologically critical processes for dinoflagellates [7,8] because they are associated with genetic recombination [5,8]; termination of HABs [8]; resistance to adverse environments [9,10]; protection from viruses, grazers, and parasite attacks [8]; and geographic expansion of populations [11,12,13]. More importantly, resting cysts can germinate into vegetative cells under suitable conditions (e.g., warm spring/summer seasons, typically in the following year) after a dormancy period, which provides “inoculation” for new rounds of population growth and even initiates HABs [7,9,14]. Therefore, surveys on germination of dinoflagellate cysts are of vital importance for monitoring and forecasting of HABs.

Due to the complexity of field environments, however, it is challenging to replicate the germination conditions of dinoflagellate cysts in laboratory settings. Significant discrepancies of cyst germination dynamics exist between the laboratory and the field, e.g., *Alexandrium* spp. [10,15,16,17]. Consequently, conducting in situ germination experiments on cysts is essential and urgent. Internationally, previous research on in situ germination of resting cysts has primarily employed two types of devices. The first type involves placing an inverted apparatus on the seabed, which is typically deployed and retrieved by divers [18,19]. However, this design presents many limitations, including instability under ocean currents, considerable difficulties in collecting germinated water samples, and a high risk of contamination from the seawater outside of the devices. The second type, notably improved by Ishikawa et al. (2007) [15], consists of a two-chamber system: sediment is placed in the lower compartment, while filtered seawater is supplied to the upper section. After assembly, the device is deployed on the seabed to enable in situ cyst germination. This design represents a significant advancement over earlier devices and has served as a foundational tool for subsequent studies in this field. Nevertheless, it still exhibits certain shortcomings, such as complex setup procedures and a lack of an adjustable counterweight to accommodate varying hydrodynamic conditions across different environments, which may compromise the stability of the device on the seabed. Moreover, to date, no studies on the in situ germination of dinoflagellate cysts have been carried out in China. Previous studies on the in situ germination of dinoflagellate cysts have largely remained unexplored. These studies focus on detecting the germination in individual species, such as *Alexandrium catenella*, *A. minutum*, *A. pacificum*, *Scrippsiella*, and *Gonyaulax*, and species identification relies primarily on microscopes [15,16,17,18,19,20,21,22,23,24,25]. Obtaining a comprehensive inventory of all germinated dinoflagellate species in a certain environment may face significant challenges due to methodological limitations.

Jiaozhou Bay, a semi-enclosed bay, is situated on the south coast of the Shandong Peninsula, China, and connects to the northwest of the South Yellow Sea through a narrow channel, with a surface area of about 374 km^2^ and an average depth of only seven meters [26]. As the earliest, most comprehensively and systematically studied marine bay in China, the bay features a densely populated coastline with intensive industrial and agricultural activities [26,27]. Marine environments of the bay are influenced by high-intensity anthropogenic activities, including urbanization, terrestrial discharges, international shipping, large-scale engineering projects, and mariculture [27,28,29]. Since the 1990s, the bay has experienced frequent HABs, making it an ideal area for studying HAB outbreaks, and historical records indicate many dinoflagellate-related HABs in the bay (e.g., *Noctiluca scintillans* and *Margalefidinium fulvescens*) [28,30,31]. Morphological and molecular identification of sediment samples has detected 63 species of dinoflagellate cysts [8,32,33,34,35], whereas identification of water samples has identified 224 dinoflagellates [26,28,36] in Jiaozhou Bay. Notably, many species in the vegetative cell checklist have never appeared in prior cyst records, and some species laboratory-confirmed to produce cysts have not yet been detected in sediments.

In this study, we implemented the recently developed Germlings Harvester (GEHA), designed for the in situ monitoring of the germination of resting-stage cells of plankton from marine sediment [37], and combined it with metabarcoding analysis to study the in situ germination of dinoflagellate resting cysts. By coupling GEHA with metabarcoding analysis targeting dinoflagellate-specific 28S rDNA primers and key environmental parameters (e.g., temperature, salinity, and dissolved oxygen), we investigated the species diversity of in situ germinated dinoflagellate cysts. This approach provides foundational technical and data-driven insights for monitoring and early warning of HABs in the study region, underscoring the significant potential of the GEHA for broader applications in HAB research and management.

## 2. Materials and Methods

### 2.1. Sampling Site

**Figure 1:**
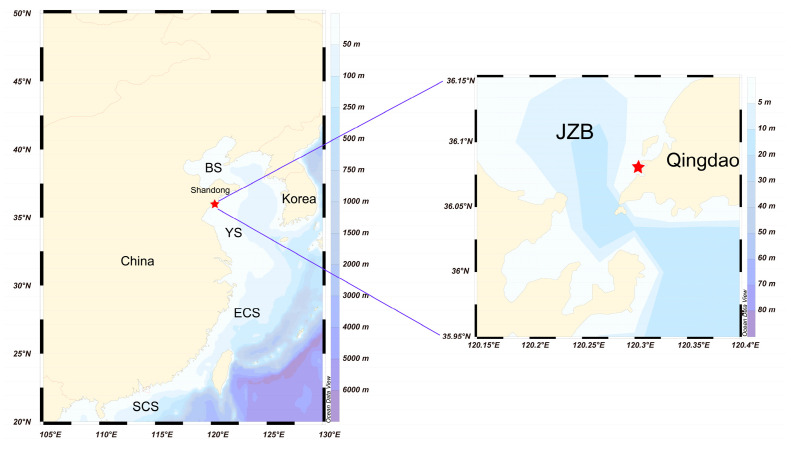
The sampling site (the closed red pentagram) in Jiaozhou Bay (120.314° E, 36.084° N). Notes: BS = Bohai Sea, YS = Yellow Sea, ECS = East China Sea, SCS = South China Sea, JZB = Jiaozhou Bay.

The in situ sampling site of the GEHA was located at the southeast coast of Jiaozhou Bay, within the Qingdao Cruise Terminal (as shown in Figure 1). The area has an average water depth of 5 m, with coordinates at 120.314° E, 36.084° N. The water exchange between the area and the open sea is effective, with a half-water-exchange period of 5 d [38]. The distribution of and variation in water temperature have obvious seasonal characteristics. The lowest water temperature appears in January (2.9 °C) and the highest in September (25.5 °C) [39]. The germination experiments were conducted from April to May 2024, during which the temperature in the area ranged between 10 and 15 °C [39]. Characterized by well-developed tourism and severe eutrophication, the area serves as a representative site for monitoring the impact of the germination of dinoflagellate cysts on HABs in Jiaozhou Bay.

### 2.2. In Situ Sampling for the Germinated Dinoflagellate Cysts with GEHA

The GEHA consists of a stainless-steel fixed bracket and four acrylic cylinders. Each cylinder features a hollow structure and is positioned symmetrically with two inlet/outlet pipes at the upper part The pipes are wrapped with double-layered 5 μm mesh nylon membranes, ensuring the exchange of seawater inside and outside the cylinders while preventing contamination by external dinoflagellates on internally germinated dinoflagellates within the cylinders. The pore size of the mesh nylon membranes can be adjusted based on water environments and the cell size of the target species. The design and manufacturing of the GEHA have been submitted for patent application.

**Figure 2:**
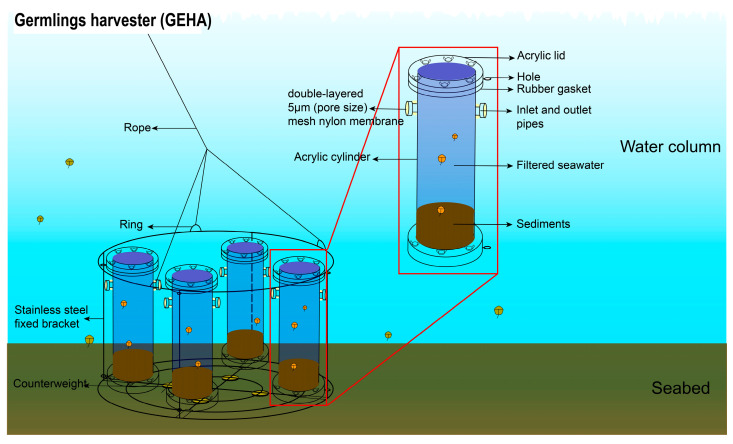
The schematic diagram of Germlings Harvester (GEHA).

The schematic diagram of the GEHA is shown in Figure 2. In situ sediment samples, collected 30 m from the GEHA monitoring site, were packed into two sets of GEHAs. Then, in situ collected seawater, filtered through a 0.22 μm pore-size filter, was poured into the cylinders. After sealing the lids, GEHAs were slowly deployed on the seabed for the germination experiments. The two sets of GEHAs were retrieved after 5 days and 20 days. Seawater samples (4 L per cylinder) from the GEHA were collected via siphoning, while, for comparison (as control), 2 L of water samples were also taken, respectively, from the surface and bottom seawater from the location where the GEHA was deployed. Additionally, the environmental conditions of the seawater, such as temperature and dissolved oxygen (DO), were measured using the YSI (Yellow Springs Instrument, Yellow Springs, OH, USA) during GEHA deployment and retrieval. All of the above structures and handling procedures of the GEHA were comprehensively detailed in Shi et al. (2025) [37].

### 2.3. DNA Extraction, PCR Amplification, and Metabarcoding Sequencing

A pair of primers (DinoF primer, 5′-KACTTTGRRAAGAGAGTTAAAW-3′; DinoR, 5′-TCYGTGTTTCAAGACGGGTC-3′) was used to amplify about 400 bases of the partial 28S rDNA sequence, including the highly variable D2 domain, intended mainly for dinoflagellates [32]. Genomic DNA of water samples, including seawater samples in the GEHA and surface and bottom seawater samples surrounding the GEHA, was extracted from 0.4 μm hydrophilic polycarbonate membranes (47 mm diameter, Merck Millipore Ltd., Darmstadt, Germany); each filtered 2 L of water samples using a plant DNA extraction kit (Tiangen, Beijing, China) according to the kit manufacturer’s protocol. Genomic DNA of the sediment samples was extracted from the resting cysts using the Fast DNA SPIN Kit for Soil (MP Biomedicals, Santa Ana, CA, USA). The resting cysts in the sediment samples were concentrated from 32 g of sediment for each sediment sample using the sodium polytungstate (SPT) protocol [40] prior to DNA extraction. The quantity and quality of total DNA were analyzed with the ND-2000 NanoDrop spectrophotometer (Thermo Fisher Scientific, Somerset, NJ, USA).

All samples were sequenced with the NovaSeq-PE250 platform by LC-Bio Technology Co., Ltd. (Hangzhou, China). Paired-end sequencing reads were demultiplexed according to sample-specific barcode information, followed by barcode and primer sequence trimming using the software Cutadapt (v1.9) [41]. Subsequently, paired-end reads were merged into contiguous tags via overlap regions using the software FLASH (v1.2.8) [42]. Sequences containing ambiguous bases (>5% N-content) or that were shorter than the quality threshold according to the fqtrim (v0.94) and chimeric sequences were filtered using the software Vsearch (v2.3.4) [43]. High-resolution amplicon sequence variants (ASVs) and abundance tables were generated through DADA2 denoising [44], with taxonomic annotations assigned against the Nucleotide database. The raw sequencing data set was submitted to the NCBI Short Read Archive (SRA) database under accession number PRJNA1302703.

### 2.4. Statistical Analyses

ASVs with above 97% identities and 100% coverage with the reference sequences in the NCBI GenBank database were annotated to species levels. ASVs with lower coverage and identity were manually checked and corrected by blasting the individual sequences in the NCBI’s BLASTn (v2.16.0) [13,32,45]. Rarefaction curves were plotted for each sample, and an unweighted hierarchical clustering tree was generated with ASVs using QIIME2 [46]. All community diversity parameters (Shannon, Chao1, Good’s coverage, and Simpson index) were calculated with QIIME2 [46]. Venn diagrams, Sankey diagrams, and principal coordinates analysis were executed using the website Tutool (http://cloudtutu.com.cn/ (accessed on 26 May 2025)). Column, line, and pie charts were generated using the Origin software (Version 2021).

## 3. Results

### 3.1. Overview for the Metabarcoding Sequencing

A total of 16 samples were subjected to metabarcoding sequencing, categorized into three groups: (1) in situ sediment samples (S); (2) samples from the GEHA capturing germinated cysts (i.e., in situ sediment germination, Sg); and (3) surface seawater (Sw) and bottom seawater (Bw) samples collected from the GEHA deployment site (see Table S1 for detailed sample information). From these samples, 1,996,087 raw sequence tags were generated, with 1,885,489 valid tags retained after quality control. A total of 2006 amplicon sequence variants (ASVs) were assigned to dinoflagellates. Good’s coverage values exceeded 99% for all samples (Table S2), and rarefaction curves approached a plateau (Figure S1), indicating sufficient sequencing depth and robust representation of the dinoflagellate assemblages in this study.

### 3.2. Species Diversity of In Situ Germinated Dinoflagellate Cysts

**Figure 3:**
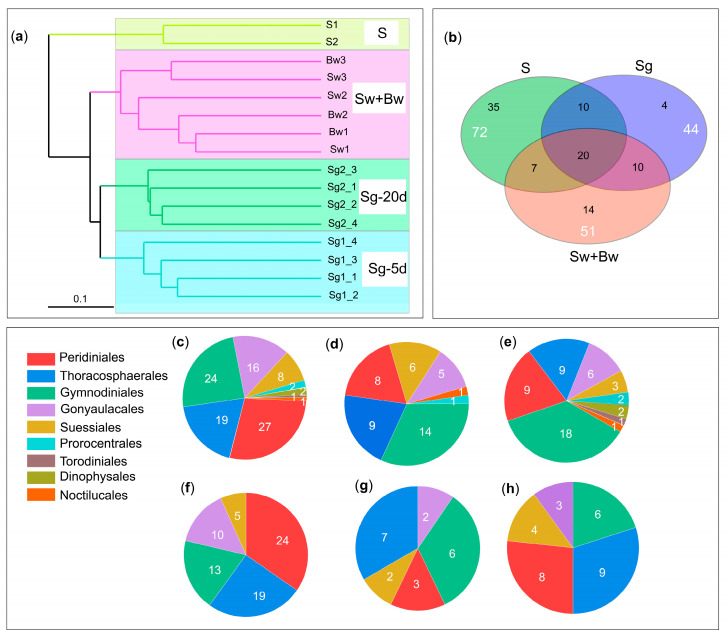
Species diversity of dinoflagellates in Sg, S, Sw+Bw. (**a**) UPGMA cluster analysis of Sg, S, Sw+Bw; (**b**) Venn diagram of Sg, S, Sw+Bw; (**c**) number of orders and corresponding species from all three groups (Sg, S, Sw+Bw); (**d**) numbers of order and corresponding species from Sg; (**e**) number of orders and corresponding species from Sw+Bw; (**f**) number of orders and corresponding species from S; (**g**) number of common orders and corresponding species shared among Sg, S, Sw+Bw; (**h**) number of common orders and corresponding species shared between Sg and S. Notes: In situ sediment germination of GEHA = Sg; surface and bottom seawater surrounding GEHA = Sw+Bw; in situ sediments = S; in situ germination period of 5 d = Sg-5 d; in situ germination period of 20 d = Sg-20 d.

The UPGMA cluster analysis clearly grouped the 16 samples into three distinct clusters (Sg, Sw+Bw, and S) (Figure 3a). The three clusters revealed significant differences among samples from different sources (i.e., water samples within the GEHA, water samples surrounding the GEHA, and sediment samples) but minor differences within samples of the same cluster in species diversity, indicating a similar community structure within Sg, Sw+Bw, and S each but greater variability among the three groups. The differences in dinoflagellate species across Sg, Sw+Bw, and S are detailed as follows: (1) Across all samples (Sg, Sw+Bw, and S), 100 dinoflagellate species were identified, belonging to 54 genera, 26 families, and 9 orders (Figure 3c, Table S3). (2) In group Sg, 44 dinoflagellate species were identified, belonging to 31 genera, 18 families, and 7 orders (Figure 3d, Table S3). (3) In group Sw+Bw, 51 dinoflagellate species were identified, belonging to 33 genera, 24 families, and 9 orders (Figure 3e, Table S3). (4) In group S, 71 dinoflagellate species were identified, belonging to 37 genera, 15 families, and 5 orders (Figure 3f, Table S3). (5) In total, 20 species were shared among all three groups, belonging to 12 genera, 9 families, and 5 orders (Figure 3b,g, Table S3). (6) In total, 30 species were shared between Sg and S, belonging to 20 genera, 12 families, and 5 orders (Figure 3b,h, Table S3), accounting for 70% of the total germinated species in group Sg.

**Figure 4:**
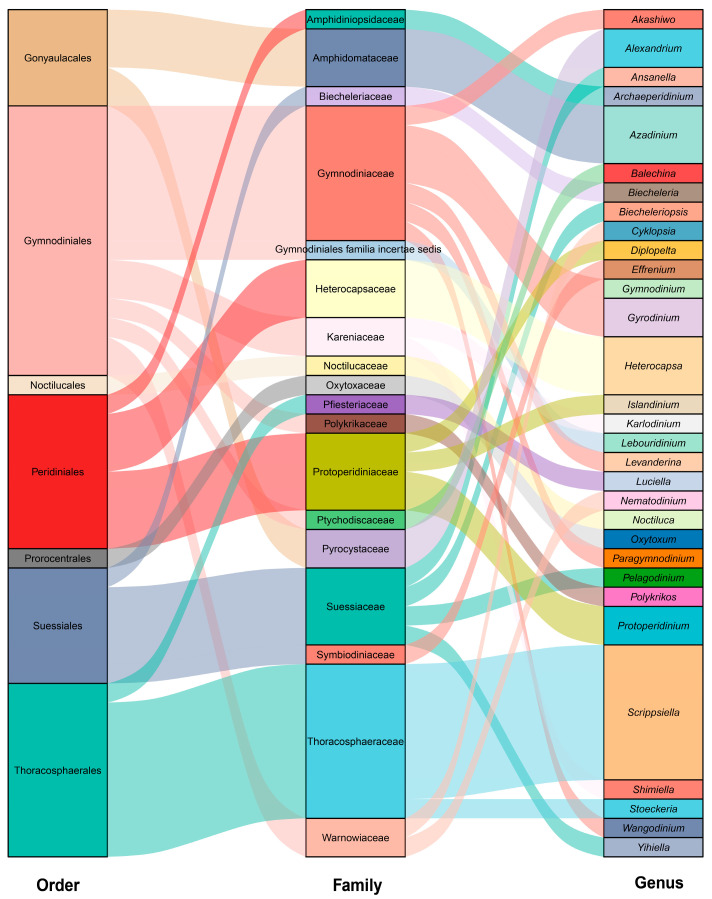
Genera, families, and orders of in situ germinated dinoflagellate cysts.

**Figure 5:**
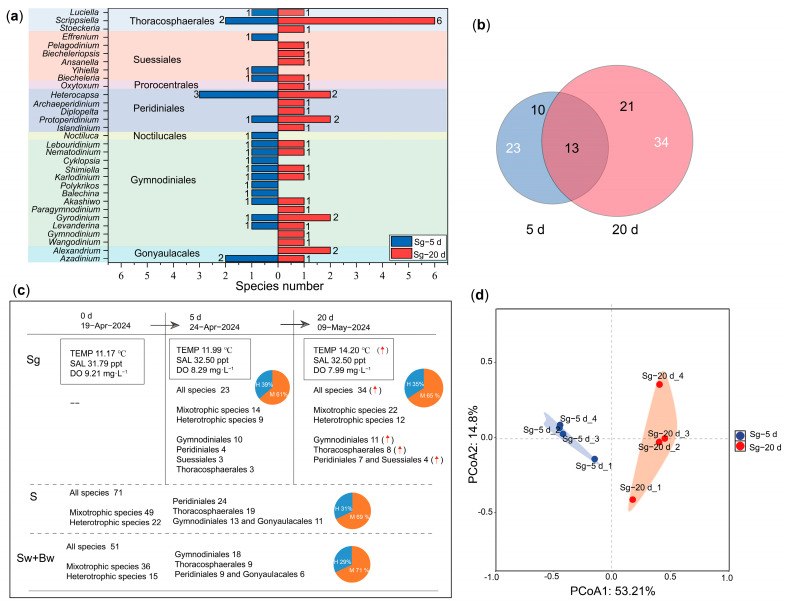
Shifts in dinoflagellate species within GEHA during in situ germination periods of 5 d (Sg-5 d) and 20 d (Sg-20 d). (**a**) Genus and corresponding species number of dinoflagellates within GEHA during in situ germination periods of 5 d and 20 d; (**b**) Venn diagram of species diversity from in situ germination periods of 5 d and 20 d; (**c**) schematic representation of dinoflagellate species and species number within GEHA and environmental conditions during in situ germination periods of 5 d and 20 d; (**d**) principal coordinates analysis (PCoA) of in situ germination periods of 5 d and 20 d. Notes: In situ sediment germination of GEHA = Sg; surface and bottom seawater surrounding GEHA = Sw+Bw; in situ sediments = S; in situ germination period of 5 d = Sg-5 d; in situ germination period of 20 d = Sg-20 d; H = heterotrophic; M = mixotrophic; TEMP = temperature; DO = dissolved oxygen; SAL = salinity; the red upward arrows indicate an increase in species number.

A total of 44 dinoflagellate species, belonging to 31 genera, 18 families, and 7 orders, were identified from in situ germination of cysts in the GEHA (Figure 4). The three most dominant orders were Gymnodiniales (14 species), Thoracosphaerales (9 species), and Peridiniales (8 species). The top four dominant families were Thoracosphaeraceae (eight species), Gymnodiniaceae (seven species), Suessiaceae (four species), and Protoperidiniaceae (four species). The three most dominant genera were Scrippsiella (seven species), Heterocapsa (three species), and Azadinium (three species) (Figure 4). A total of 19 species had cells larger than 15 μm, 28 species exceeded 10 μm, 42 species were larger than 5 μm, and 2 species were approximately 5 μm (Table S4). After 5 days of in situ germination (Sg-5 d), 23 dinoflagellate species were identified, spanning 19 genera, 15 families, and 6 orders. The order Gymnodiniales was the most diverse, with 10 species, followed by Peridiniales (4 species), Suessiales (3 species), Thoracosphaerales (3 species), Gonyaulacales (2 species), and Noctilucales (1 species) (Figure 5a, Table S3). Among these, 9 species were strictly heterotrophic, and 14 species were mixotrophic (Figure 5c, Table S5). After 20 days of in situ germination (Sg-20 d), 34 dinoflagellate species were identified, encompassing 25 genera, 14 families, and 6 orders. The order Gymnodiniales again exhibited the highest diversity with 11 species, followed by Thoracosphaerales (8 species), Peridiniales (7 species), Suessiales (4 species), Gonyaulacales (3 species), and Prorocentrales (1 species) (Figure 5a, Table S3). Of these, 12 species were strictly heterotrophic, and 22 species were mixotrophic (Figure 5c, Table S5). A total of 13 species, representing 12 genera, 9 families, and 4 orders, were shared between the 5-day (Sg-5d) and 20-day (Sg-20d) in situ germination samples (Figure 5a,b, Table S3).

During the 5-day in situ germination period (Sg-5 d), temperature increased from 11.17 °C to 11.99 °C, dissolved oxygen declined from 9.21 mg/L to 8.29 mg/L, and salinity rose from 31.79 ppt to 32.50 ppt (Figure 5c). During the in situ 20-day germinating period (Sg-20 d), temperature rose to 14.2 °C, dissolved oxygen dropped to 7.99 mg/L, and salinity was 32.50 ppt (Figure 5c). Principal coordinates analysis (PCoA) clearly grouped the eight in situ germinated samples into two distinct clusters, with the first principal coordinate axis accounting for more than 50% of the variance (Figure 5d). Both PCoA and UPGMA cluster analysis revealed significant differences in species diversity between those germinated in 5 days (Sg-5 d) and those in 20 days (Sg-20 d), which also indicated a highly similar community structure within the samples incubated for the same period of time (Figure 3a and Figure 5d). From Sg-5 d to Sg-20 d, the number of germinated dinoflagellate species increased by 11 species and six genera (Figure 5a,c). Specifically, the order Thoracosphaerales increased from three to eight species, with the genus *Scrippsiella* contributing four species; Peridiniales increased from four to seven species; and Gymnodiniales, Suessiales, and Prorocentrales each gained one species (Figure 5a). Additionally, the number of HAB-causing species increased from 7 to 10, while the number of corresponding orders changed from four to three (Table S3). The ratio of mixotrophic to heterotrophic species remained stable (approximately 2:1) across germination durations (Sg-5 d vs. Sg-20 d), closely mirroring the ratio in the community of sediment and seawater outside the GEHA (Figure 5c, Table S5).

### 3.3. Species of Particular Importance from In Situ Germinated Dinoflagellate Cysts

Among the 44 in situ germinated dinoflagellate species, 12 were HAB-causing species, including *Azadinium polongum*, *Azadinium poporum*, *Alexandrium leei*, *Alexandrium pacificum*, *Levanderina fissa*, *Akashiwo sanguinea*, *Karlodinium veneficum*, *Lebouridinium glaucum*, *Noctiluca scintillans*, *Stoeckeria algicida*, *Scrippsiella acuminata*, and *Luciella masanensis* (Table 1). Of these, six species (*Azadinium poporum*, *Alexandrium leei*, *Alexandrium pacificum*, *Akashiwo sanguinea*, *Karlodinium veneficum*, and *Stoeckeria algicida*) are known to be toxic (Table 1). In addition, five species were identified as novel cyst producers, including the widely distributed HAB-causing species *Noctiluca scintillans*, *Balechina gracilis*, *Cyklopsia gemma*, *Gyrodinium* cf. *spirale*, and *Nematodinium parvum* (Table 1). Among the 39 species previously reported to produce cysts, 5 were observed for the first time in this study to germinate from marine sediments: HAB-causing species *Stoeckeria algicida*, the symbiotic dinoflagellate *Effrenium voratum*, *Scrippsiella lachrymose*, *Scrippsiella* aff. *acuminata*, and *Yihiella yeosuensis* (Table 1).

## 4. Discussion

### 4.1. GEHA Combined with Metabarcoding Analysis Detected High Dinoflagellate Diversity from In Situ Germination

The challenges associated with in situ germination studies have historically limited research, with previous investigations relying primarily on morphological identification, resulting in the documentation of only nine dinoflagellate species from in situ germination: *Scippsiella* sp. [23], *Ensiculifera carinata*, *Gonyaulax spinifera*, *Gonyaulax verior*, *Protoperidinium claudicans*, *Protoperidinium conicoides*, *Protoperidinium conicum* [20], *Alexandrium catenella* [16,17], and *Alexandrium pacificum* [17]. Therefore, we developed the GEHA, a novel in situ germination device, for the in situ monitoring of the germination of resting-stage cells of plankton [37]. In this study, we employed the GEHA, combined with metabarcoding analysis targeting 28S rDNA-specific primers for dinoflagellates, to identify 44 dinoflagellate species from in situ germinated cysts, spanning 31 genera, 18 families, and 7 orders. Compared to 51 and 71 dinoflagellates identified in surface/bottom seawater surrounding the GEHA (Sw+Bw) and in situ sediments samples (S), respectively, the lower species richness in the GEHA samples (Sg) may be attributed to three factors: (1) Variable germination requirements, as different dinoflagellate cysts have distinct optimal germination temperatures [100,101], potentially leading to lower germination rates for species like *Scrippsiella acuminata* [102], which was abundant in sediments but less prevalent in GEHA. (2) “Dominance bias” in high-throughput sequencing, which may obscure low-abundance species [103,104]. Notably, dominant species, such as the winter bloom-causing dinoflagellate *Heterocapsa rotundata* [105] in the GEHA and the HAB-causing dinoflagellate *Levanderina fissa* in the surrounding seawater, contrasted with higher species evenness in sediment samples, mitigating the masking of rare species. (3) Due to the differences between sediment and seawater samples, we employed specific DNA extraction kits (as described in the Materials and Methods) to maximize DNA extraction efficiency. For sediment samples, the majority of cells obtained after SPT treatment were resting-stage cells, which generally have thick cell walls that are difficult to break using conventional kits. Therefore, we used a DNA extraction kit specifically designed for sediments. For water samples, since algal cells were filtered onto polycarbonate membranes, the sediment-specific kit could not fully dissolve the membranes. Hence, we adopted a DNA extraction kit containing organic solvents for membrane dissolution. Two specialized kits were employed to maximize the DNA extraction from all environmental samples, which may have influenced the observed species divergence. In addition to DNA extraction, for factors such as sequencing depth, limitations in genetic marker resolution, and bioinformatic parameters, we maintained consistency across all samples. Crucially, UPGMA clustering and PCoA of the Sg, Sw+Bw, and S groups, as well as the Sg-5d and Sg-20d samples, revealed distinct community structures inside and outside the GEHA, further validating its reliability for in situ germination monitoring.

**Figure 6:**
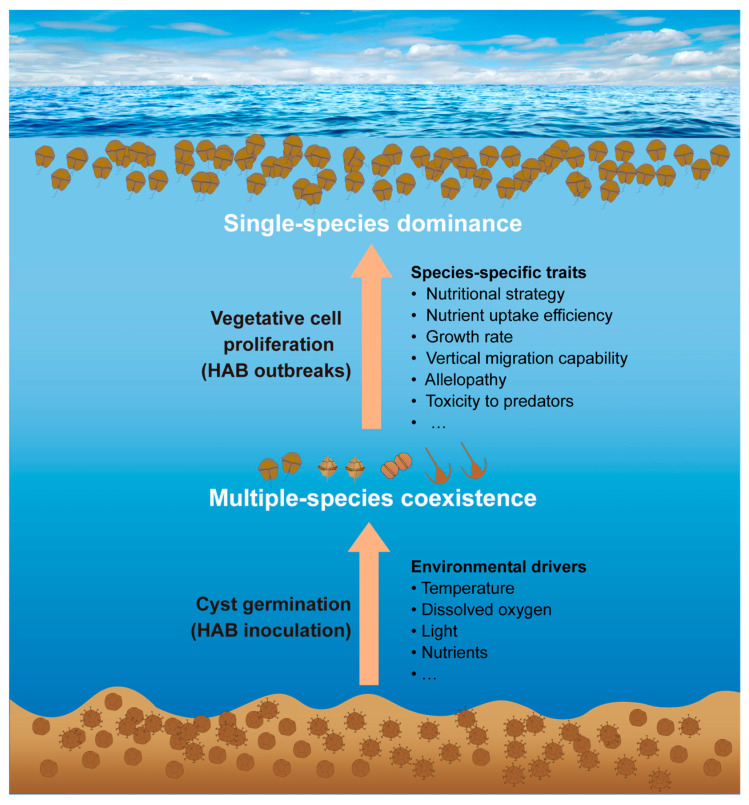
A conceptual diagram showing the ecological processes and environmental drivers for outbreaks of dinoflagellate HABs that initiate from resting cyst germination.

Notably, the consistent ratio (approximately 2:1) of mixotrophic to heterotrophic dinoflagellates in the samples of in situ sediments and seawater outside GEHA, as well as across germination durations (Sg-5 d vs. Sg-20 d), further demonstrates that the germination condition within the GEHA does resemble the in situ scenario (i.e., the GEHA did not significantly alter the genuine field condition). As Barrie Dale noted in a recent interview [106], “Cysts indicate potential bloom initiation size but not bloom magnitude or fate, as water-column factors (e.g., currents, nutrients, predation, competition) dominate outcomes.” Therefore, the in situ cyst germination by the GEHA further solidifies our findings (i.e., many HAB-causing species can germinate in the field) and logically proves which species produce cysts and whether/when these cysts can germinate in a certain ecosystem setting; together, this proves the existence of a “seed source” for the vegetative cell population and possibility of HAB outbreaks under favorable growth conditions. However, as noted above, the scale or fate of HABs is influenced by a combination of environmental factors and species-specific traits (Figure 6). In this study, dinoflagellates were classified into mixotrophic and strictly heterotrophic categories. Theoretically, dinoflagellates exhibit three primary modes of nutrition, including autotrophy, heterotrophy, and mixotrophy [107,108]. According to previous studies, at least half of dinoflagellates are strictly heterotrophs [109,110]. In contrast, autotrophy may exist only theoretically; in practice, Lin et al. (2022) [111] have demonstrated that most dinoflagellates capable of autotrophy are mixotrophic because most (or all) dinoflagellate species lack functional *metE* genes with an N-terminal domain or do not have *metE* at all. Consequently, we classified dinoflagellate nutritional modes into mixotrophy and strict heterotrophy, without separating autotrophy as an independent category.

The use of 5 μm mesh nylon membranes in the GEHA introduces a potential limitation, as the two species with cell sizes of approximately 5 μm (*Paragymnodinium asymmetricum* [86] and *Biecheleriopsis adriatica* [63]) may have originated from external seawater rather than in situ sediment germination. However, given the double-layered 5 μm nylon mesh design and that *B. adriatica* is known to produce resting cysts [63,86], their presence in the GEHA most likely originated from in situ germination, though the external possibility cannot be completely ruled out. Furthermore, we conducted a blank control experiment for the GEHA by deploying it with only 0.22 μm of filtered seawater filled in it (i.e., no sediment introduced) on the seabed for 14 d. Subsequent microscopic examination of the water retrieved from the device revealed no cell debris or species larger than 5 μm. Therefore, among the 44 species detected from in situ germination in the GEHA, except for *Paragymnodinium asymmetricum* and *Biecheleriopsis adriatica*, the 5 μm mesh nylon membrane should prevent all the other 42 species (with sizes > 5 μm) from crossing the “baffle”, and hence, they were most likely germinated in situ within the GEHA.

### 4.2. Many Dinoflagellates of Particular Ecological Importance Germinated In Situ from Marine Sediments

Our results demonstrated that a few HAB-causing and toxic species that were proven as cyst producers in the laboratory after 2010 (e.g., *Akashiwo sanguinea* [50], *Karlodinium veneficum* [74], *Azadinium polongum* [60], and *Azadinium poporum* [61]) do produce resting cysts in the field and can also germinate in the field, which logically implies the possible role played by these cysts in seeding (or initiating) the HABs of these species. Notably, *Karlodinium veneficum* produces a suite of ichthyotoxic polyketides known as karlotoxins [75], which induce gill epithelial damage and osmoregulatory failure [112,113], and consequently, *K. veneficum* blooms are frequently associated with mass mortality events of aquatic organisms [76,114]. *Azadinium poporum* is known for the production potential of AZA (azaspiracid toxins) and is widely distributed in coastal waters in China [61]. Blooms of *Akashiwo sanguinea* have caused mass mortalities of invertebrates and fish [115,116], as well as seabirds [117], while concurrently incurring substantial economic losses to aquaculture via ecosystem disruption [118]. Additionally, *Stoeckeria algicida* has not been shown to have a specific toxin, though it has been confirmed to cause mortality in fish and other marine microalgae [97,119,120]. The cyst germination of *S. algicida* from sediments was reported for the first time in this study, which has only been observed in laboratory cultures previously [97]. It is noteworthy that the HAB-causing species *Noctiluca scintillans*, detected in the GEHA, has caused multiple HAB events in temperate, subtropical, and tropical coastal waters worldwide [84,121,122] and has also caused many serious HABs in Jiaozhou Bay [28]. Although *Noctiluca scintillans* have not been reported to produce toxins directly lethal to marine life or pose indirect threats to human health, the high biomass accumulation during its blooms causes significant physical damage to marine ecosystems, including gill clogging in fish and invertebrates [123], and light attenuation, leading to seagrass/coral photosynthesis suppression [124]. *N. scintillans* has not been reported to produce cysts but was detected from in situ germination within the GEHA, which provides direct evidence not only for the production of resting cysts but also for the capacity of the cysts to germinate from natural sediments. Furthermore, five species were identified as novel cyst producers, and five others were documented for the first time as germinating from marine sediment cysts, offering robust field evidence for their cyst-forming and germination capabilities.

The in situ germination of *E. voratum*, a symbiotic dinoflagellate, is particularly significant. Our previous study [66] first reported this species’ production of resting cysts through asexual reproduction and described cyst germination in clonal cultures, making *E. voratum* the first reported cyst-producing species in the family Symbiodiniaceae. However, the germination of *E. voratum* cysts has not been observed in marine sediments, and it remains doubtful whether the cysts can germinate in situ. This study provides the first evidence of in situ germination of *E. voratum* cysts from marine sediments, highlighting their ecological importance to coral reef ecosystems and their potential relevance to global climate change.

### 4.3. Species Diversity of In Situ Germinated Dinoflagellate Cysts with Germination Duration Extended

Over the 5-day (Sg-5 d) and 20-day (Sg-20 d) germination periods, significant differences in temperature and dissolved oxygen (*t*-tests, *p* < 0.05) were observed, while no significant difference was observed in salinity (*t*-tests, *p* > 0.05). Concurrently, germinated dinoflagellate species increased from 23 to 34, suggesting that temperature, dissolved oxygen, and germination duration are highly likely to be key drivers of cyst germination diversity. Previous studies have shown that cyst germination is regulated by both internal and external factors [7]. After the completion of mandatory dormancy, cyst germination occurs when environmental conditions become favorable [125]. Thus, environmental factors, such as temperature, light, dissolved oxygen, and nutrients, can influence cyst germination. The impact of these factors varies significantly across different cyst types: (1) For temperature, Anderson and Morel (1979) [14] combined field observations with laboratory experiments and found that increasing temperatures enhanced the germination rates of both field-collected cysts and laboratory-stored *Alexandrium tamarense* cysts after four months of storage. Accoroni et al. (2015) [126] found that the water temperature threshold of 25 °C played a key role in the germination of *Ostreopsis* cf. *ovate* cysts. (2) For light, Anderson et al. (1987) [127] found *Gonyaulax polyedra* cysts required light to germinate, while cysts of *Alexandrium tamarense*, *Scrippsiella* sp., and *Gonyaulax verior* exhibited 5 to 20 times higher germination rates under light conditions compared to darkness. In contrast, *Gonyaulax rugosum* showed high germination rates under both light and dark conditions. (3) For dissolved oxygen, cysts of *Alexandrium tamarense* and *Alexandrium catenella* required a certain level of dissolved oxygen for germination, and germination was completely inhibited when dissolved oxygen levels dropped to 0.01 mg/L [128]. (4) For nutrients, when dinoflagellate cysts in surface sediments were exposed to a culture environment containing organic phosphorus, their germination rates increased significantly [100]. However, Wang et al. (2010) [129] found that nutrient concentrations had no effect on the germination of *Scrippsiella acuminata* cysts. Beyond environmental factors, some studies have reported that phytohormones (e.g., abscisic acid, gibberellic acid, and melatonin) may also play vital roles in regulating cyst germination [130,131]. However, most research has been conducted in controlled laboratory settings, which struggle to replicate complex field conditions. There are only a few relevant studies, such as Natsuike et al. (2017) [17], on the germination dynamics of *Alexandrium catenella* and *A. pacificum*, and Ishikawa et al. (2022) [21,22], on the germination dynamics of *Chattonella* and centric diatoms, that focus on individual species germination fluxes and rely on microscopic examination. Our study, utilizing the GEHA with environmental monitoring and molecular identification, demonstrated that the temperature, dissolved oxygen, and germination duration within the observed ranges of variations may have all contributed to the elevated number of species detected in the GEHA, but other factors (e.g., nutrients, light, and hydrodynamic scenarios) might be also responsible for the observations, and all the abovementioned factors should be definitely measured in future investigations that use the GEHA to monitor, in situ, the seasonal dynamics of cyst germination in the field. Therefore, much more in situ field monitoring work is required in different environmental settings, which is exactly what our approach was developed for.

Additionally, we found the number of HAB-causing species increased from 7 to 10, while the number of corresponding orders changed from four to three with extended germination duration. These results indicated that the duration of cyst germination within GEHA and environmental factors appeared to have influenced the germination and possibly growth of HAB-causing species. However, due to the relatively short time period of this study, the germination of HAB-causing species also requires further investigation under different conditions, such as seasonal dynamics and fluctuations under diverse marine environments. Long-term continual monitoring of cyst germination is essential for establishing a framework of early warning and forecasting models of HABs, which will be the focus of our future research. While we did not observe that HAB-causing species are more likely to germinate after longer incubation, we analyzed whether the presence or absence of thecal plates was associated with the major germinating taxa. The analysis revealed that unarmored dinoflagellates accounted for 52% of the germinated taxa, whereas they accounted for only 32% of the taxa in the sediment. The number of unarmored dinoflagellate species in the germinated water within the GEHA was higher than that in the sediment, which suggests that unarmored dinoflagellates seem more likely to germinate under natural conditions, but this needs more extensive, convincing observations. Additionally, we found that cysts did not exhibit a clear tendency for which nutritional mode is more conducive to germination in the field, as the proportions of mixotrophic and heterotrophic dinoflagellates were similar in both the sediment and the germinated water within the GEHA. It is worth further investigating which types of cysts are more likely to germinate in the field, such as those with varying cyst wall thickness, different nutritional modes, and the presence or absence of thecal plates. We also noticed that those orders and families that produce thick-walled cysts and/or are the most speciose taxa were more detected from the GEHA (e.g., Gymnodiniales, Thoracosphaerales, and Peridiniales). Moreover, most of the species detected from the GEHA were also those commonly detected in marine sediments. These results demonstrate the reliability and authenticity of the GEHA for in situ cyst germination, laying a solid foundation for future long-term, continual research.

## 5. Conclusions

By integrating the Germlings Harvester (GEHA) with metabarcoding analysis and environmental monitoring, this study identified 44 dinoflagellate species that germinated in situ from marine sediments in Jiaozhou Bay, China. This included 12 species associated with HABs, 6 toxic species, 5 novel cyst producers, and 5 species with newly documented germination from sediment cysts. Additionally, the number of germinated species increased by 11 by extending the germination duration from 5 to 20 days, and the consistent ratio (approximately 2:1) of mixotrophic to heterotrophic dinoflagellates was observed in samples of in situ sediments and seawater outside the GEHA, as well as across germination durations (Sg-5 d vs. Sg-20 d). These findings establish a robust technical framework combining the GEHA, molecular identification, and in situ environmental monitoring (e.g., temperature and nutrients) in long-term in situ monitoring studies, demonstrating significant potential for predicting and providing early warnings of HAB dynamics. Furthermore, this approach shows promise for broader applications across diverse taxonomic groups in freshwater, estuarine, and marine ecosystems.

## Figures and Tables

**Table 1:** The 44 identified dinoflagellate species that were germinated in situ from resting cysts within GEHA and the relevant information.

Species	Synonyms	HAB Effects	Was It the Resting Cyst Formation Reported Before?	Was Germination of Resting Cysts Collected from the Marine Sediment Reported Before?	Is It Heterotrophic?	References
*Akashiwo sanguinea*	*Gymnodinium sanguineum*	B/T	Y	Y		[47,48,49,50,51]
*Alexandrium leei*	*Protogonyaulax leei*	B/T	Y	Y		[52,53]
*Alexandrium pacificum*		B/T	Y	Y		[17,54,55]
*Ansanella granifera*			Y	Y		[32,37,56]
*Archaeperidinium saanichi*			Y	Y	Y	[57]
*Azadinium cuneatum*			Y	Y		[58]
*Azadinium polongum*		B	Y	Y		[37,59,60]
*Azadinium poporum*		B/T	Y	Y		[61]
*Balechina gracilis*	*Gymnodinium gracile*				Y	[62]
*Biecheleria brevisulcata*			Y	Y		[63]
*Biecheleriopsis adriatica*			Y	Y		[63]
*Cyklopsia gemma*					Y	[64]
*Diplopelta pusilla*	*Lebouraia pusilla*		Y	Y	Y	[65]
*Effrenium voratum*	*Symbiodinium voratum*		Y			[66]
*Gymnodinium smaydae*			Y	Y		[37,67]
*Gyrodinium* cf. *spirale*					Y	[68,69]
*Gyrodinium heterogrammum*			Y	Y	Y	[13,37,69]
*Heterocapsa pseudotriquetra*			Y	Y		[37,70]
*Heterocapsa rotundata*			Y	Y		[71,72]
*Heterocapsa steinii*			Y	Y		[71]
*Islandinium tricingulatum*	*Protoperidinium tricingulatum*		Y	Y	Y	[73]
*Karlodinium veneficum*		B/T	Y	Y		[74,75,76]
*Lebouridinium glaucum*	*Katodinium glaucum*	B	Y	Y	Y	[37,77]
*Levanderina fissa*	*Gyrodinium instriatum*	B	Y	Y		[78,79]
*Luciella masanensis*		B	Y	Y	Y	[37,80,81,82]
*Nematodinium parvum*	*Warnowia parva*					[83]
*Noctiluca scintillans*	*Noctiluca pacifica*	B			Y	[84]
*Oxytoxum lohmannii*	*Amphidinium crissum*; *Oxytoxum longum*		Y	Y	Y	[37,85]
*Paragymnodinium* *asymmetricum*			Y	Y		[37,86]
*Pelagodinium beii*	*Gymnodinium bei*		Y	Y		[37,87]
*Polykrikos kofoidii*			Y	Y	Y	[68,88]
*Protoperidinium americanum*	*Peridinium americanum*		Y	Y	Y	[73]
*Protoperidinium parthenopes*			Y	Y	Y	[73]
*Scrippsiella acuminata*	*Scrippsiella trochoidea*	B	Y	Y		[89,90,91]
*Scrippsiella* aff. *acuminata*			Y			[92]
*Scrippsiella bicarinata*			Y	Y		[32,93]
*Scrippsiella* cf. *acuminata*			Y	Y		[32]
*Scrippsiella* cf. *erinaceus*			Y	Y		[94]
*Scrippsiella donghaiensis*			Y	Y		[63]
*Scrippsiella lachrymosa*			Y			[95]
*Shimiella gracilenta*			Y	Y	Y	[37,96]
*Stoeckeria algicida*		B/T	Y		Y	[97]
*Wangodinium sinense*			Y	Y		[98]
*Yihiella yeosuensis*			Y			[99]

Notes: B = harmful agal bloom-causing species; T = toxic species (all toxic species are harmful agal bloom-causing species); Y = yes.

## Data Availability

The data are contained within the article and the Supplementary Materials.

## Supplementary Materials

The following supporting information can be downloaded at https://www.mdpi.com/article/10.3390/life15111670/s1: Figure S1: Rarefaction curve of metabarcoding sequencing. Table S1: The sampling name, sampling sites, and time of 16 samples. Table S2: The community diversity parameters in the samples. Table S3: All species identified from in situ sediments (S), in situ sediment germination of GEHA (Sg), bottom seawater (Bw), and surface seawater (Sw) surrounding GEHA. Table S4: Cell size of the 44 identified dinoflagellates from in situ cyst germination in GEHA. Table S5: Mixotrophic and heterotrophic species in the samples of in situ sediments (S) and surface and bottom seawater surrounding GEHA (Sw+Bw), as well as across the 5 d (Sg-5 d) and 20 d (Sg-5 d) in situ germination periods. References [52,55,57,58,60,61,62,63,64,65,69,70,73,80,83,84,86,87,89,95,96,97,98,99,132,133,134,135,136,137,138,139,140,141,142,143,144,145] are cited in the supplementary materials.
