# Supplementary material for: In Situ Harvesting and Molecular Identification for the Germinating Species Diversity of Dinoflagellate Resting Cysts in Jiaozhou Bay, China
Source: Life (Basel). 2025 Oct 27;15(11):1670. doi: 10.3390/life15111670 (PMC12653938; doi:10.3390/life15111670)
Supplement: Supplementary file 1 [file life-15-01670-s001.zip › life-3913630-supplementary.pdf]

Supplementary materials

In-situ harvesting and molecular identification for the germinating species diversity of dinoflagellate resting cysts in Jiaozhou Bay, China

Shuo Shi[1,3], Wanli Yang[1,3], Zhe Tao[1,6], Fengting Li[1,2], Ben Wei[1,4], Caixia Yue[1,5], Yunyan Deng[1,2], Lixia Shang[1,2], Zhaoyang Chai[1,2*], Ying Zhong Tang[1,2*]

[1] CAS Key Laboratory of Marine Ecology and Environmental Sciences, Institute of Oceanology, Chinese Academy of Sciences, Qingdao 266071, China

[2] Laboratory for Marine Ecology and Environmental Science, Qingdao Marine Science and Technology Center, Qingdao 266237, China

[3] University of Chinese Academy of Sciences, Beijing 100049, China

[4] Qingdao Innovation and Development Center, Harbin Engineering University, Qingdao, 266000, China

[5] Animal, Plant and Food Inspection Center of Nanjing Customs, Nanjing 210000, China

[6] Shandong Key Laboratory of Marine Ecological Restoration, Shandong Marine Resource and Environment Research Institute, Yantai 264006, China

[*] Corresponding authors: yingzhong.tang@qdio.ac.cn (YZT); zhaoyangchai@qdio.ac.cn (ZC).

Contents of the Supplementary materials:

Figure S1. Rarefaction curve of metabarcoding sequencing.

Table S1. The sampling name, sampling sites, and time of 16 samples.

Table S2. The community diversity parameters in the samples.

Table S3. All species identified from in-situ sediments (S), in-situ sediment germination of GEHA (Sg), bottom seawater (Bw) and surface seawater (Sw) surrounding GEHA.

Table S4. Cell size of 44 identified dinoflagellates from in-situ cysts germination of GEHA.

Table S5. Mixotrophic and heterotrophic species in the samples of in-situ sediments (S), and seawater outside GEHA (Sw+Bw), as well as across 5 d (Sg-5 d) and 20d (Sg-5 d) in-situ germination period.

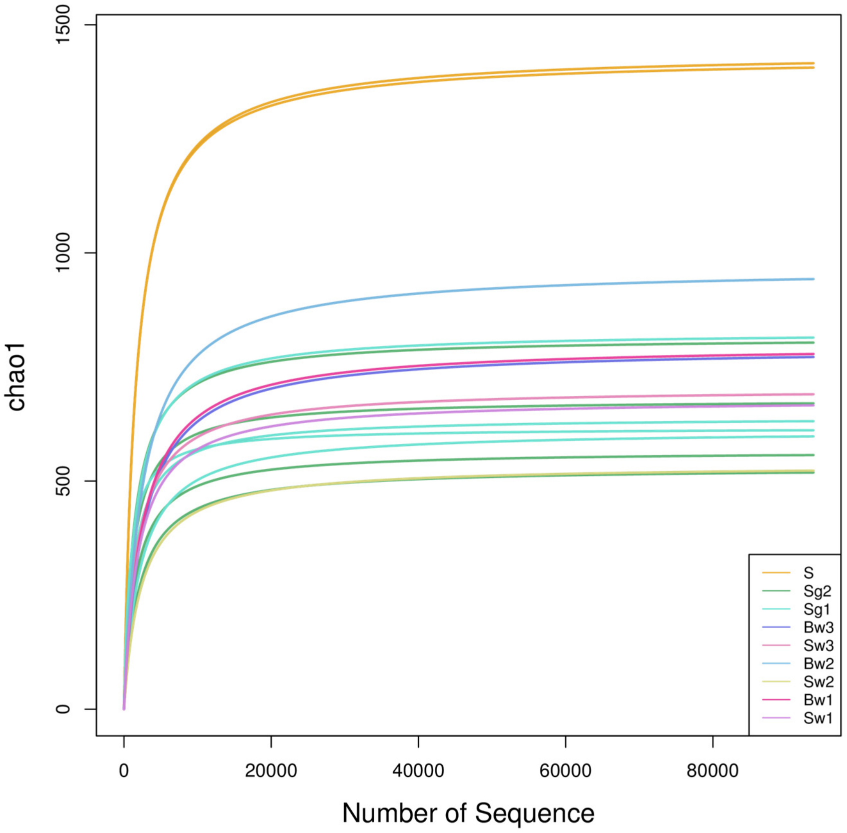

Figure S1. Rarefaction curve of metabarcoding sequencing. Note: The sampling names correspond to the names in Table S1.

Table S1. The sampling name, sampling sites, and time of 16 samples.

Sampling name	Sampling sites	Time
S1; S2	In-situ sediments (S)	19-Apr-2024
Sg1_1; Sg1_2; Sg1_3; Sg1_4	In-situ sediment germination (Sg-5 d)	24-Apr-2024
Sg2_1; Sg2_2; Sg2_3; Sg2_4	In-situ sediment germination (Sg-20 d)	09-May-2024
Sw1	Surface seawater (Sw-0 d)	19-Apr-2024
Sw2	Surface seawater (Sw-5 d)	24-Apr-2024
Sw3	Surface seawater (Sw-20 d)	09-May-2024
Bw1	Bottom seawater (Bw-0 d)	19-Apr-2024
Bw2	Bottom seawater (Bw-5 d)	24-Apr-2024
Bw3	Bottom seawater (Bw-20 d)	09-May-2024

Table S2. The community diversity parameters in the samples.

Sampling name	Shannon	Simpson	Chao1	Goods coverage
S1; S2	6.90	0.95	1386.37	1.00
Sg1_1; Sg1_2; Sg1_3; Sg1_4	6.00	0.94	655.63	1.00
Sg2_1; Sg2_2; Sg2_3; Sg2_4	5.64	0.93	629.08	1.00
Sw1	5.50	0.93	656.22	1.00
Sw2	5.05	0.92	512.04	1.00
Sw3	5.56	0.94	685.66	1.00
Bw1	5.46	0.93	759.76	1.00
Bw2	5.82	0.95	926.76	1.00
Bw3	4.77	0.88	762.13	1.00

Note: The sampling names correspond to the names in Table S1.

Table S3. All species identified from in-situ sediments (S), in-situ sediment germination of GEHA (Sg), bottom seawater (Bw) and surface seawater (Sw) surrounding GEHA.

**Order**	**Family**	**Genus**	**Species**	**Synonyms**	**S**	**Sg-20d**	**Sg-5d**	**Bw-20d**	**Sw-20d**	**Bw-5d**	**Sw-5d**	**Bw-0d**	**Sw-0d**	**Is it heterotrophic?**
		Dinophysis	*Dinophysis acuminata*					+	+	+	+	+	+	Y
Dinophysales	Dinophysaceae	*Phalacroma*	*Phalacroma rotundatum*					+		+	+			Y
			Azadinium polongum		+		+	+	+		+	+		
			Azadinium cuneatum		+		+		+	+	+	+	+	
			Azadinium dalianense		+			+	+	+		+	+	
			Azadinium galwayense		+									
			Azadinium zhuanum		+									
	Amphidomataceae	*Azadinium*	*Azadinium poporum*			+								
	Ceratiaceae	*Tripos*	*Tripos lineatus*	*Neoceratium lineatum*									+	
			Gonyaulax digitale		+									
	Gonyaulacaceae	*Gonyaulax*	*Gonyaulax spinifera*	*Spiniferites ramosus*							+		+	
	Lingulodiniaceae	*Lingulaulax*	*Lingulaulax polyedra*	*Gonyaulax polyedra,Lingulodinium polyedra*	+									
			Alexandrium leei	*Protogonyaulax leei*	+	+								
			Alexandrium affine	*Protogonyaulax affinis*	+									
			Alexandrium catenella							+	+		+	
			Alexandrium ostenfeldii	*Alexandrium peruvianum*	+									
Gonyaulacales	Pyrocystaceae	*Alexandrium*	*Alexandrium pacificum*			+								
Gymnodiniales		*Wangodinium*	*Wangodinium sinense*		+	+			+					

Order	Family	Genus	Species	Synonyms	S	Sg-20d	Sg-5d	Bw-20d	Sw-20d	Bw-5d	Sw-5d	Bw-0d	Sw-0d	Is it heterotrophic?
		Pseudocochlodinium	*Pseudocochlodinium profundisulcus*	*Cochlodinium geminatum ,Polykrikos geminatus*	+									
			Gymnodinium aureolum	*Gyrodinium aureolum*				+	+	+	+	+	+	
			Gymnodinium smaydae		+	+		+	+	+				
			Gymnodinium impudicum	*Gyrodinium impudicum*	+									
			Gymnodinium microreticulatum		+									
			Gymnodinium inusitatum		+								+	
			Gymnodinium trapeziforme		+									
	Gymnodiniaceae	*Gymnodinium*	*Gymnodinium catenatum*		+									
		Levanderina	*Levanderina fissa*	*Gyrodinium instriatum*	+	+	+	+	+	+	+	+	+	
			Gyrodinium cf. *spirale*			+		+	+	+			+	Y
			Gyrodinium heterogrammum			+	+	+	+	+	+	+	+	Y
		Gyrodinium	*Gyrodinium rubrum*							+				Y
		Lepidodinium	*Lepidodinium chlorophorum*					+	+	+	+	+	+	
		Paragymnodinium	*Paragymnodinium asymmetricum*		+	+		+						
		Akashiwo	*Akashiwo sanguinea*	*Gymnodinium sanguineum*		+	+	+	+	+	+	+	+	
	Ptychodiscaceae	*Balechina*	*Balechina gracilis*	*Gymnodinium gracile*			+	+		+		+	+	Y
	Polykrikaceae	*Pheopolykrikos*	*Pheopolykrikos hartmannii*	*Polykrikos hartmannii*	+									
		Polykrikos	*Polykrikos kofoidii*		+		+		+	+		+	+	Y
	Kareniaceae	*Karlodinium*	*Karlodinium veneficum*			+	+	+	+	+		+	+	

Order	Family	Genus	Species	Synonyms	S	Sg-20d	Sg-5d	Bw-20d	Sw-20d	Bw-5d	Sw-5d	Bw-0d	Sw-0d	Is it heterotrophic?
		Shimiella	*Shimiella gracilenta*			+	+	+	+	+		+	+	Y
		Cyklopsia	*Cyklopsia gemma*				+			+	+	+	+	Y
	Warnowiaceae	*Nematodinium*	*Nematodinium parvum*	*Warnowia parva*	+	+	+	+	+	+	+	+	+	
	Gymnodiniales familia incertae sedis	*Lebouridinium*	*Lebouridinium glaucum*	*Katodinium glaucum*		+	+	+	+	+	+	+	+	Y
Noctilucales	Noctilucaceae	*Noctiluca*	*Noctiluca scintillans*	*Noctiluca pacifica*			+			+	+	+	+	Y
			Ensiculifera mexicana		+									
			Ensiculifera carinata		+									
		Ensiculifera	*Ensiculifera tyrrhenica*	*Pentapharsodinium tyrrhenicum,Peridinium tyrrhenicum*	+									
	Ensiculiferaceae	*Pentapharsodinium*	*Pentapharsodinium dalei*	*Peridinium dalei*	+					+	+		+	
			Islandinium minutum		+									Y
		Islandinium	*Islandinium tricingulatum*	*Protoperidinium tricingulatum*	+	+								Y
		Selenopemphix	*Selenopemphix undulata*		+									Y
			Protoperidinium americanum	*Peridinium americanum*	+	+								Y
			Protoperidinium concavum	*Peridinium concavum*	+									Y
			Protoperidinium lewisiae		+									Y
			Protoperidinium monovelum		+					+	+	+	+	Y
		Protoperidinium	*Protoperidinium parthenopes*		+	+	+							Y
Peridiniales	Protoperidiniaceae	*Diplopelta*	*Diplopelta pusilla*	*Lebouraia pusilla*	+	+								Y

Order	Family	Genus	Species	Synonyms	S	Sg-20d	Sg-5d	Bw-20d	Sw-20d	Bw-5d	Sw-5d	Bw-0d	Sw-0d	Is it heterotrophic?
		Diplopsalis	*Diplopsalis lenticula*		+									Y
		Oblea	*Oblea rotunda*		+									Y
		Preperidinium	*Preperidinium meunieri*		+									Y
		Niea	*Niea chinensis*		+									Y
		Qia	*Qia lebouriae*	*Diplopsalis lenticula var. lebouriae Nie*	+									Y
			Archaeperidinium minutum		+			+						Y
			Archaeperidinium saanichi		+	+								Y
	Amphidiniopsidaceae	*Archaeperidinium*	*Archaeperidinium constrictum*		+									Y
			Heterocapsa iwatakii					+						
			Heterocapsa lanceolata					+						
			Heterocapsa rotundata		+	+	+	+	+	+	+	+	+	
			Heterocapsa pseudotriquetra		+	+	+			+	+	+		
	Heterocapsaceae	*Heterocapsa*	*Heterocapsa steinii*		+		+	+	+	+	+	+	+	
	Kryptoperidiniaceae	*Kryptoperidinium*	*Kryptoperidinium triquetrum*	*Heterocapsa triquetra*					+					
	Oxytoxaceae	*Oxytoxum*	*Oxytoxum lohmannii*	*Amphidinium crassum*; *Oxytoxum longum*		+		+	+	+		+		Y
Prorocentrales	Prorocentraceae	*Prorocentrum*	*Prorocentrum triestinum*						+					
	Biecheleriaceae	*Biecheleria*	*Biecheleria brevisulcata*		+	+	+	+	+	+	+	+	+	
	Borghiellaceae	*Dactylodinium*	*Dactylodinium arachnoides*							+	+	+	+	
Suessiales	Suessiaceae	*Yihiella*	*Yihiella yeosuensis*		+		+							

Order	Family	Genus	Species	Synonyms	S	Sg-20d	Sg-5d	Bw-20d	Sw-20d	Bw-5d	Sw-5d	Bw-0d	Sw-0d	Is it heterotrophic?
			Ansanella granifera			+								
		Ansanella	*Ansanella catalana*		+									
		Biecheleriopsis	*Biecheleriopsis adriatica*		+	+								
		Pelagodinium	*Pelagodinium beii*	*Gymnodinium bei*	+	+		+	+					
	Symbiodiniaceae	*Effrenium*	*Effrenium voratum*	*Symbiodinium voratum*			+							
		Tintinnophagus	*Tintinnophagus acutus*		+									Y
		Stoeckeria	*Stoeckeria algicida*		+	+								Y
		Amyloodinium	*Amyloodinium ocellatum*	*Oodinium ocellatum*	+									Y
		Leonella	*Leonella granifera*	*Thoracosphaera granifera*	+									
		Posoniella	*Posoniella tricarinelloides*		+									
			Scrippsiella acuminata	*Scrippsiella trochoidea*	+	+		+	+					
			Scrippsiella aff. *acuminata*		+	+	+			+	+	+	+	
			Scrippsiella bicarinata		+	+			+					
			Scrippsiella cf. *acuminata*		+	+								
			Scrippsiella cf. *erinaceus*		+		+				+		+	
			Scrippsiella sweeneyae		+									
			Scrippsiella donghaiensis		+	+		+	+	+	+	+	+	
			Scrippsiella enormis		+									
			Scrippsiella infula		+									
Thoracosphaerales	Thoracosphaeraceae	*Scrippsiella*	*Scrippsiella kirschiae*		+									

Order	Family	Genus	Species	Synonyms	S	Sg-20d	Sg-5d	Bw-20d	Sw-20d	Bw-5d	Sw-5d	Bw-0d	Sw-0d	Is it heterotrophic?
			Scrippsiella lachrymosa		+	+					+		+	
			Scrippsiella precaria		+					+	+		+	
			Scrippsiella trochoidea var. aciculifera		+						+			
	Pfiesteriaceae	*Luciella*	*Luciella masanensis*		+	+	+	+	+	+				Y
Torodiniales	Torodiniaceae	*Torodinium*	*Torodinium robustum*					+		+			+	

Notes: S = in-situ sediments; Sg-5 d = in situ germination periods of 5 d within GEHA; Sg-20 d = in situ germination periods of 20 d within GEHA; Sw-0 d, 5 d, 20 d = 0 d, 5 d, 20 d of surface seawater surrounding GEHA; Bw-0 d, 5 d, 20 d = 0 d, 5 d, 20 d of bottom seawater surrounding GEHA; Y = Yes.

Table S4. Cell size of the 44 identified dinoflagellates from in-situ cysts germination of GEHA.

Species	Synonyms	Cell size		References
Noctiluca scintillans	*Noctiluca pacifica*	0.2-2 mm in diameter		[1]
Balechina gracilis	*Gymnodinium gracile*	75-140 µm long, 45-70 µm wide		[2]
Archaeperidinium saanichi		40.7-55.1 µm long, 38.6-49.0 µm wide		[3]
Cyklopsia gemma		approximately 40 µm long, 25 µm wide		[4]
Diplopelta pusilla	*Lebouraia pusilla*	30.0-40.0 µm		[5]
Akashiwo sanguinea	*Gymnodinium sanguineum*	30-35 µm long, 25-29 µm wide		[6]
Alexandrium leei	*Protogonyaulax leei*	30-45 µm long, 25-40 µm wide		[7]
Protoperidinium americanum	*Peridinium americanum*	25-40 µm long, 25-35 µm wide		[8]
Protoperidinium parthenopes		25-35 µm long, 25-32 µm wide		[8]
Gyrodinium heterogrammum		32-35 µm long, 24-30 µm wide	>15 µm	[9]
Alexandrium pacificum		20-39.5 µm long, 22-44 µm wide		[10]
Gyrodinium cf. *spirale*		110-130 µm long, 20-30 µm wide		[11]
Islandinium tricingulatum	*Protoperidinium tricingulatum*	20-30 µm long, 20-25 µm wide		[8]
Polykrikos kofoidii		30-55 µm long, 20-35 µm wide		[12]
Scrippsiella lachrymosa		34-44 µm long, 18-32 µm wide		[13]
Scrippsiella aff. *acuminata*		20-27 µm long, 17-25 µm wide		[14]
Levanderina fissa	*Gyrodinium instriatum*	21.9-49.4 µm long, 16.5-34.8 µm wide		[15]
Nematodinium parvum	*Warnowia parva*	25.8-38.6 µm long, 16-25.1 µm wide		[16]
Oxytoxum lohmannii	*Amphidinium crassum*	approximately 22 by 16 µm in size		[17]
Scrippsiella acuminata	*Scrippsiella trochoidea*	17-23 µm long, 14-20 µm wide		[18]
Scrippsiella donghaiensis		17-48 µm long, 14-30 µm wide		[14]
Lebouridinium glaucum	*Katodinium glaucum*	35-40 µm long, 14-22 µm wide		[19]
Heterocapsa pseudotriquetra		18.4-27.2 µm long, 14.4-21.6 µm wide		[20]
Heterocapsa steinii		17.8-25.9 µm long, 13.0-17.6 µm wide	>10 µm	[21]
Scrippsiella cf. *erinaceus*		15-28 µm long, 13-22 µm wide		[22]
Scrippsiella bicarinata		17-35 µm long, 13-31 µm wide		[23]
Scrippsiella cf. *acuminata*		15-28.1 µm long, 11.3-25 µm wide		[24]
Stoeckeria algicida		14.4-20.8 µm long, 10.0-17.4 µm wide		[25]
Heterocapsa rotundata		9.0-11.7 µm long, 8.7-10 µm wide		[26]
Ansanella granifera		10-15.0 µm long, 8.5-12.4 µm wide		[27]

Karlodinium veneficum		9-18 μm long, 8-14 μm wide	>5 μm	[28]
Azadinium cuneatum		11.2-16.9 μm long, 8.3-12.7 μm wide		[29]
Luciella masanensis		9.6-15.7 μm long, 7.8-12.7 μm wide		[30]
Azadinium polongum		10-17 μm long, 7-14 μm wide		[31]
Azadinium poporum		9.9-17.1 μm long, 7.6-12.9 μm wide		[32]
Pelagodinium beii	*Gymnodinium bei*	8.8-11.4 μm long, 6.0-7.5 μm wide		[33]
Wangodinium sinense		8.5-11.8 μm long, 6.2-9.3 μm wide		[34]
Biecheleria brevisulcata		7.2-9.2 μm long, 5.8-8.9 μm wide		[35]
Effrenium voratum	*Symbiodinium voratum*	7.3-12.7 μm long, 5.52-11.4 μm wide		[36]
Shimiella gracilenta		9-15 μm long, 5-9 μm wide		[37]
Yihiella yeosuensis		7.9-10.6 μm long, 5.0-7.0 μm wide		[38]
Gymnodinium smaydae		6.3-10.9 μm long, 5.1-10.0 μm wide		[39]
Paragymnodinium asymmetricum		7.9-12.6 μm long, 4.7-9.0 μm wide	≈5 μm	[40]
Biecheleriopsis adriatica		5.8-10.1 μm long, 4.1-8.0 μm wide		[35]

Table S5. Mixotrophic and heterotrophic species in the samples of in-situ sediments (S), and Surface and bottom seawater surrounding GEHA (Sw+Bw), as well as across 5 d (Sg-5 d) and 20d (Sg-5 d) in-situ germination period.

	In-situ sediment (S)	Seawater within GEHA-5 d (Sg-5 d)	Seawater within GEHA-20 d (Sg-20 d)	Surface and bottom seawater surrounding GEHA (Sw+Bw)
Total species number	71	23	34	51
	-	*Akashiwo sanguinea*	*Akashiwo sanguinea*	*Akashiwo sanguinea*
Mixotrophy	*Alexandrium affine*	-	-	-
	-	-	-	*Alexandrium catenella*
	Alexandrium leei	-	*Alexandrium leei*	-
	-	-	*Alexandrium pacificum*	-
	Alexandrium ostenfeldii	-	-	-
	Ansanella catalana	-	-	-

	-	-	*Ansanella granifera*	-
	Azadinium cuneatum	*Azadinium cuneatum*	-	*Azadinium cuneatum*
	Azadinium dalianense	-	-	*Azadinium dalianense*
	Azadinium galwayense	-	-	-
	Azadinium zhuanum	-	-	-
	-	-	*Azadinium poporum*	-
	Azadinium polongum	*Azadinium polongum*	-	*Azadinium polongum*
	Biecheleria brevisulcata	*Biecheleria brevisulcata*	*Biecheleria brevisulcata*	*Biecheleria brevisulcata*
	Biecheleriopsis adriatica	-	*Biecheleriopsis adriatica*	-
	-	-	-	*Dactylodinium arachnoides*
	Ensiculifera tyrrhenica	-	-	-
	Ensiculifera carinata	-	-	-
	Ensiculifera mexicana	-	-	-
	-	*Effrenium voratum*	-	-
	-	-	-	*Gonyaulax spinifera*
	Gonyaulax digitale	-	-	-
	-	-	-	*Gymnodinium aureolum*
	Gymnodinium catenatum	-	-	-
	Gymnodinium impudicum	-	-	-
	Gymnodinium inusitatum	-	-	*Gymnodinium inusitatum*
	Gymnodinium microreticulatum	-	-	-
	Gymnodinium smaydae	-	*Gymnodinium smaydae*	*Gymnodinium smaydae*
	Gymnodinium trapeziforme	-	-	-
	-	-	-	*Heterocapsa iwatakii*
	-	-	-	*Heterocapsa lanceolata*
	Heterocapsa pseudotriquetra	*Heterocapsa pseudotriquetra*	*Heterocapsa pseudotriquetra*	*Heterocapsa pseudotriquetra*
	Heterocapsa rotundata	*Heterocapsa rotundata*	*Heterocapsa rotundata*	*Heterocapsa rotundata*

	Heterocapsa steinii	*Heterocapsa steinii*	-	*Heterocapsa steinii*
	-	*Karlodinium veneficum*	*Karlodinium veneficum*	*Karlodinium veneficum*
	-	-	-	*Kryptoperidinium triquetrum*
	-	-	-	*Lepidodinium chlorophorum*
	Leonella granifera	-	-	-
	Levanderina fissa	*Levanderina fissa*	*Levanderina fissa*	*Levanderina fissa*
	Lingulaulax polyedra	-	-	-
	Nematodinium parvum	*Nematodinium parvum*	*Nematodinium parvum*	*Nematodinium parvum*
	Paragymnodinium asymmetricum	-	*Paragymnodinium asymmetricum*	*Paragymnodinium asymmetricum*
	Pelagodinium beii	-	*Pelagodinium beii*	*Pelagodinium beii*
	Pentapharsodinium dalei	-	-	*Pentapharsodinium dalei*
	Pheopolykrikos hartmannii	-	-	-
	Posoniella tricarinelloides	-	-	-
	Pseudocochlodinium profundisulcus	-	-	-
	-	-	-	*Prorocentrum triestinum*
	Scrippsiella acuminata	-	*Scrippsiella acuminata*	*Scrippsiella acuminata*
	Scrippsiella aff. acuminata	*Scrippsiella aff. acuminata*	*Scrippsiella aff. acuminata*	*Scrippsiella aff. acuminata*
	Scrippsiella bicarinata	-	*Scrippsiella bicarinata*	*Scrippsiella bicarinata*
	Scrippsiella cf. acuminata	-	*Scrippsiella cf. acuminata*	-
	Scrippsiella cf. erinaceus	*Scrippsiella cf. erinaceus*	-	*Scrippsiella cf. erinaceus*
	Scrippsiella donghaiensis	-	*Scrippsiella donghaiensis*	*Scrippsiella donghaiensis*
	Scrippsiella enormis	-	-	-
	Scrippsiella infula	-	-	-
	Scrippsiella kirschiae	-	-	-
	Scrippsiella lachrymosa	-	*Scrippsiella lachrymosa*	*Scrippsiella lachrymosa*
	Scrippsiella precaria	-	-	*Scrippsiella precaria*

	Scrippsiella sweeneyae	-	-	-
	Scrippsiella trochoidea var. aciculifera	-	-	*Scrippsiella trochoidea var. aciculifera*
	-	-	-	*Torodinium robustum*
	-	-	-	*Tripos lineatus*
	Wangodinium sinense	-	*Wangodinium sinense*	*Wangodinium sinense*
	Yihiella yeosuensis	*Yihiella yeosuensis*	-	-
Mixotrophy: total species	**49/71=69%**	**14/23=61%**	**22/34=65%**	**36/51=71%**
Heterotrophy	*Amyloodinium ocellatum*	-	-	-
	Archaeperidinium minutum	-	-	*Archaeperidinium minutum*
	Archaeperidinium constrictum	-	-	-
	Archaeperidinium saanichi	-	*Archaeperidinium saanichi*	-
	-	*Cyklopsia gemma*	-	*Cyklopsia gemma*
	-	-	-	*Dinophysis acuminata*
	Diplopelta pusilla	-	*Diplopelta pusilla*	-
	Diplopsalis lenticula	-	-	-
	-	*Gymnodinium gracile*	-	*Gymnodinium gracile*
	-	*Gyrodinium heterogrammum*	*Gyrodinium heterogrammum*	*Gyrodinium heterogrammum*
	-	-	*Gyrodinium cf. spirale*	*Gyrodinium cf. spirale*
	-	-	-	*Gyrodinium rubrum*
	Islandinium minutum	-	-	-
	Islandinium tricingulatum	-	*Islandinium tricingulatum*	-
	-	*Lebouridinium glaucum*	*Lebouridinium glaucum*	*Lebouridinium glaucum*
	Luciella masanensis	*Luciella masanensis*	*Luciella masanensis*	*Luciella masanensis*
	-	*Noctiluca scintillans*	-	*Noctiluca scintillans*
	Niea chinensis	-	-	-

	Oblea rotunda	-	-	-
	-	-	-	-
	-	-	*Oxytoxum lohmannii*	*Oxytoxum lohmannii*
	Polykrikos kofoidii	*Polykrikos kofoidii*	-	*Polykrikos kofoidii*
	-	-	-	*Phalacroma rotundatum*
	Preperidinium meunieri	-	-	-
	Protoperidinium americanum	-	*Protoperidinium americanum*	-
	Protoperidinium concavum	-	-	-
	Protoperidinium lewisiae	-	-	-
	Protoperidinium monovelum	-	-	*Protoperidinium monovelum*
	Protoperidinium parthenopes	*Protoperidinium parthenopes*	*Protoperidinium parthenopes*	-
	Qia lebouriae	-	-	-
	Selenopemphix undulata	-	-	-
	-	*Shimiella gracilenta*	*Shimiella gracilenta*	*Shimiella gracilenta*
	Stoeckeria algicida	-	*Stoeckeria algicida*	-
	Tintinnophagus acutus	-	-	-
Heterotrophy: total species	**22/71=31%**	**9/23=39%**	**12/34=35%**	**15/51=29%**
Mixotrophy: Heterotrophy	**2.22**	**1.55**	**1.83**	**2.4**

References

1. Li, X.; Lu, W.; Jiang, F.; Chen, Z.; Chang, Y.; Wang, Z.; Yan, T.; Li, J.; Wang, W.; Chen, X. The dinoflagellate *Noctiluca scintillans* in China: a review of its distribution and role in harmful algal blooms. *Mar. Pollut. Bull.* **2023**, *194*, 115415.
2. Gomez, F.; Artigas, L.F.; Gast, R.J. Molecular phylogeny and synonymy of *Balechina gracilis* comb. nov. (=*Gymnodinium gracile*), a widespread polymorphic unarmored dinoflagellate (Dinophyceae). *J. Phycol.* **2021**, *57*, 694–697.
3. Mertens, K.N.; Yamaguchi, A.; Kawami, H.; Ribeiro, S.; Leander, B.S.; Price, A.M.; Pospelova, V.; Ellegaard, M.; Matsuoka, K. *Archaeperidinium saanichi* sp. nov.: a new species based on morphological variation of cyst and theca within the *Archaeperidinium minutum* jörgensen 1912 species complex. *Mar. Micropaleontol.* **2012**, *96–97*, 48–62.
4. Cooney, E.C.; Holt, C.C.; Jacko-Reynolds, V.K.L.; Leander, B.S.; Keeling, P.J. Photosystems in the eye-like organelles of heterotrophic warnowiid dinoflagellates. *Curr. Biol.* **2023**, *33*, 4252-4260.e3.
5. Liu, T.; Mertens, K.N.; Gu, H. Cyst-theca relationship and phylogenetic positions of the diplopsalioideans (Peridiniales, Dinophyceae), with description of *Niea* and *Qia gen. nov. Phycologia* **2015**, *54*, 210–232.
6. Koening, M.L.; Flores Montes, M.J.; Eskinazi Leca, E.; Tiburcio, A.S.X.S. New record of *Akashiwo sanguinea* (Dinophyta) in the tropical estuarine waters of Northeastern Brazil (Western Atlantic). *Braz. J. Biol.* **2014**, *74*, 191–198.
7. Gu, H.; Zeng, N.; Liu, T.; Yang, W.; Mueller, A.; Krock, B. Morphology, toxicity, and phylogeny of *Alexandrium* (Dinophyceae) species along the coast of China. *Harmful Algae* **2013**, *27*, 68–81.
8. Liu, T.; Gu, H.; Mertens, K.N.; Lan, D. New dinoflagellate species *Protoperidinium haizhouense* sp. nov. (Peridiniales, Dinophyceae), its cyst-theca relationship and phylogenetic position within the *Monovela* group. *Phycol. Res.* **2014**, *62*, 109–124.
9. Larsen, J. Unarmoured dinoflagellates from Australian waters II. Genus *Gyrodinium* (Gymnodiniales, Dinophyceae). *Phycologia* **1996**, *35*, 342–349.
10. John, U.; Litaker, R.W.; Montresor, M.; Murray, S.; Brosnahan, M.L.; Anderson, D.M. Formal

revision of the *Alexandrium tamarense* species complex (Dinophyceae) taxonomy: the introduction of five species with emphasis on molecular-based (rDNA) classification. *Protist* **2014**, *165*, 779–804.

11. Rene, A.; Camp, J.; Garces, E. Diversity and phylogeny of Gymnodiniales (Dinophyceae) from the NW Mediterranean Sea revealed by a morphological and molecular approach. *Protist* **2015**, *166*, 234–263.
12. Nagai, S.; Matsuyama, Y.; Takayama, H.; Kotani, Y. Morphology of *Polykrikos kofoidii* and *P. schwartzii* (Dinophyceae, Polykrikaceae) cysts obtained in culture. *Phycologia* **2002**, *41*, 319–327.
13. Lewis, J. Cyst-theca relationships in *Scrippsiella* (Dinophyceae) and related Orthoperidinioid genera. *Bot. Mar.* **1991**, *34*, 91–106.
14. Gu, H.; Sun, J.; Kooistra, W.H.C.F.; Zeng, R. Phylogenetic position and morphology of thecae and cysts of *Scrippsiella* (Dinophyceae) species in the East China Sea. *J. Phycol.* **2008**, *44*, 478–494.
15. Moestrup, O.; Hakanen, P.; Hansen, G.; Daugbjerg, N.; Ellegaard, M. On *Levanderina fissa* gen. & comb. nov. (Dinophyceae) (syn. *Gymnodinium fissum*, *Gyrodinium instriatum*, *Gyr. uncatenum*), a dinoflagellate with a very unusual sulcus. *Phycologia* **2014**, *53*, 265–292.
16. García-Portela, M.; Moestrup ,Øjvind; Daugbjerg ,Niels; Altenburger ,Andreas; and Lundholm, N. Studies on the complex Warnowiaceae (dinophyceae) I. Lohmann's *Pouchetia parva* refound and renamed *Nematodinium parvum* comb. nov. (= *Warnowia parva*). *Phycologia* **2023**, *62*, 421–435.
17. Cooney, E.C.; Jacobson, D.M.; Wolfe, G.V.; Bright, K.J.; Saldarriaga, J.F.; Keeling, P.J.; Leander, B.S.; Strom, S.L. Morphology, behavior, and phylogenomics of *Oxytoxum lohmannii*, dinoflagellata. *J. Eukaryotic Microbiol.* **2024**, *71*.
18. Kretschmann, J.; Elbraezchter, M.; Zinssmeister, C.; Soehner, S.; Kirsch, M.; Kusber, W.-H.; Gottschling, M. Taxonomic clarification of the dinophyte *Peridinium acuminatum* Ehrenb., *Scrippsiella acuminata*, comb. nov. (Thoracosphaeraceae, Peridiniales). *Phytotaxa* **2015**, *220*, 239–256.
19. Gomez, F.; Takayama, H.; Moreira, D.; Lopez-Garcia, P. Unarmoured dinoflagellates with a small hyposome: *Torodinium* and *Lebouridinium* gen. nov for *Katodinium glaucum*

(Gymnodiniales, Dinophyceae). *Eur. J. Phycol.* **2016**, *51*, 226–241.

20. Iwataki, M.; Hansen, G.; Sawaguchi, T.; Hiroishi, S.; Fukuyo, Y. Investigations of body scales in twelve *Heterocapsa* species (Peridiniales, Dinophyceae), including a new species *H. pseudotriquetra* sp. nov. *Phycologia* **2004**, *43*, 394–403.
21. Tillmann, U.; Hoppenrath, M.; Gottschling, M.; Kusber, W.-H.; Elbraechter, M. Platepattern clarification of the marine dinophyte *Heterocapsa triquetra* sensu Stein (Dinophyceae) collected at the Kiel Fjord (Germany). *J. Phycol.* **2017**, *53*, 1305–1324.
22. Kretschmann, J.; Zinssmeister, C.; Gottschling, M. Taxonomic clarification of the dinophyte *Rhabdosphaera erinaceus* KAMPTNER, *Scrippsiella erinaceus* comb. nov (Thoracosphaeraceae, Peridiniales). *Syst. Biodivers.* **2014**, *12*, 393–404.
23. Zinssmeister, C.; Soehner, S.; Kirsch, M.; Facher, E.; Meier, K.J.S.; Keupp, H.; Gottschling, M. Same but different: Two novel bicarinate species of extant calcareous dinophytes (Thoracosphaeraceae, Peridiniales) from the Mediterranean Sea. *J. Phycol.* **2012**, *48*, 1107–1118.
24. Nunes, C.C. da S.; Silva, D.M.L. da; Affe, H.M. de J.; Nunes, J.M. de C. Occurrence and distribution of *Scrippsiella* cf. *acuminata* (Dinophyta, Thoracospharaceae) in a tropical estuarine gradient. *Rodriguésia* **2022**, *73*.
25. Jeong, H.J.; Kim, J.S.; Park, J.Y.; Kim, J.H.; Kim, S.; Lee, I.; Lee, S.H.; Ha, J.H.; Yih, W.H. *Stoeckeria algicida* n. gen., n. sp (Dinophyceae) from the coastal waters off southern Korea: morphology and small subunit ribosomal DNA gene sequence. *J. Eukaryotic Microbiol.* **2005**, *52*, 382–390.
26. Hansen, G. Analysis of the thecal plate pattern in the dinoflagellate *Heterocapsa rotundata* (Lohmann) comb. nov. (=*Katodinium rotundatum* (Lohmann) Loeblich). *Phycologia* **1995**, *34*, 166–170.
27. Jeong, H.J.; Jang, S.H.; Moestrup, O.; Kang, N.S.; Lee, S.Y.; Potvin, E.; Noh, J.H. *Ansanella granifera* gen. et sp. nov (Dinophyceae), a new dinoflagellate from the coastal waters of Korea. *Algae* **2014**, *29*, 75–99.
28. Bergholtz, T.; Daugbjerg, N.; Moestrup, O.; Fernández-Tejedor, M. On the identity of *Karlodinium veneficum* and description of *Karlodinium armiger* sp. nov (Dinophyceae), based on light and electron microscopy, nuclear-encoded LSU rDNA, and pigment

composition. *J. Phycol.* **2006**, *42*, 170–193.

29. Tillmann, U.; Gottschling, M.; Nezan, E.; Krock, B.; Bilien, G. Morphological and molecular characterization of three new *Azadinium* species (Amphidomataceae, Dinophyceae) from the Irminger Sea. *Protist* **2014**, *165*, 417–444.
30. Mason, P.L.; Litaker, R.W.; Jeong, H.J.; Ha, J.H.; Reece, K.S.; Stokes, N.A.; Park, J.Y.; Steidinger, K.A.; Vandersea, M.W.; Kibler, S.; et al. Description of a new genus of *Pfesteria*-like dinoflagellate, *Luciella* gen. nov (Dinophyceae), including two new species: *Luciella masanensis* sp. nov and *Luciella atlantis* sp. nov. *J. Phycol.* **2007**, *43*, 799–810.
31. Tillmann, U.; Soehner, S.; Nézan, E.; Krock, B. First record of the genus *Azadinium* (Dinophyceae) from the shetland islands, including the description of *Azadinium polongum* sp. nov. *Harmful Algae* **2012**, *20*, 142–155.
32. Gu, H.; Luo, Z.; Krock, B.; Witt, M.; Tillmann, U. Morphology, phylogeny and azaspiracid profile of *Azadinium poporum* (Dinophyceae) from the China sea. *Harmful Algae* **2013**, *21–22*, 64–75.
33. Siano, R.; Montresor, M.; Probert, I.; Not, F.; de Vargas, C. *Pelagodinium* gen. nov and *P. beii* comb. nov., a dinoflagellate symbiont of planktonic foraminifera. *Protist* **2010**, *161*, 385–399.
34. Tao, Z.; Yue, C.; Liu, Y.; Shi, S.; Li, R.; Chai, Z.; Deng, Y.; Shang, L.; Hu, Z.; Gu, H.; et al. A new ribo-type of *Wangodinium sinense* from germination of resting cysts isolated from ballast tank sediments of incoming ships to China. *J. Mar. Sci. Eng.* **2025**, *13*, 942.
35. Hu, Z.; Song, X.; Wang, J.; Tao, Z.; Sun, Y.; Li, Y.; Liu, Y.; Deng, Y.; Shang, L.; Chai, Z.; et al. Reviving and characterizing three species of dinoflagellate cysts dormant for about 70 years in the East China Sea: *Biecheleria brevisulcata, Biecheleriopsis adriatica*, and *Scrippsiella donghaienis*. *J. Oceanol. Limnol.* **2022**, *40*, 2292–2311.
36. Kang, N.S.; Kim, E.S.; Lee, J.A.; Kim, K.M.; Kwak, M.S.; Yoon, M.; Hong, J.W. First report of the dinoflagellate genus *Effrenium* in the east sea of korea: morphological, genetic, and fatty acid characteristics. *Sustainability* **2020**, *12*, 3928.
37. Ok, J.H.; Jeong, H.J.; Lee, S.Y.; Park, S.A.; Noh, J.H. *Shimiella* gen. nov. and *Shimiella gracilenta* sp. nov. (Dinophyceae, Kareniaceae), a Kleptoplastidic Dinoflagellate from Korean Waters and its Survival under Starvation. *J. Phycol.* **2021**, *57*, 70–91.
38. Jang, S.H.; Jeong, H.J.; Moestrup, O.; Kang, N.S.; Lee, S.Y.; Lee, K.H.; Seong, K.A. *Yihiella*

yeosuensis gen. Et sp. nov. (Suessiaceae, Dinophyceae), a novel dinoflagellate isolated from the coastal waters of Korea. *J. Phycol.* **2017**, *53*, 131–145.

39. Kang, N.S.; Jeong, H.J.; Moestrup, O.; Lee, S.Y.; Lim, A.S.; Jang, T.Y.; Lee, K.H.; Lee, M.J.; Jang, S.H.; Potvin, E.; et al. *Gymnodinium smaydae* n. sp., a new planktonic phototrophic dinoflagellate from the coastal waters of western Korea: morphology and molecular characterization. *J. Eukaryotic Microbiol.* **2014**, *61*, 182–203.

40. Yokouchi, K.; Takahashi, K.; Nguyen, V.N.; Iwataki, M.; Horiguchi, T. Ultrastructure and systematics of two new species of dinoflagellate, *Paragymnodinium asymmetricum* sp. nov. and *Paragymnodinium inerme* sp. nov. (Gymnodiniales, Dinophyceae). *J. Phycol.* **2020**, *56*, 730–746.
